# Extract from *Aronia melanocarpa* L. Berries Protects Against Cadmium-induced Lipid Peroxidation and Oxidative Damage to Proteins and DNA in the Liver: A Study using a Rat Model of Environmental Human Exposure to this Xenobiotic

**DOI:** 10.3390/nu11040758

**Published:** 2019-03-31

**Authors:** Magdalena Mężyńska, Małgorzata M. Brzóska, Joanna Rogalska, Anna Galicka

**Affiliations:** 1 Department of Toxicology, Medical University of Bialystok, Adama Mickiewicza 2C street, 15-222 Bialystok, Poland; joanna.rogalska@umb.edu.pl; 2 Department of Medical Chemistry, Medical University of Bialystok, Adama Mickiewicza 2A, 15-222 Bialystok, Poland; angajko@umb.edu.pl

**Keywords:** *Aronia melanocarpa* berries extract, cadmium, lipid peroxidation, liver, metallothionein, protection, protein oxidation, 8-hydroxy-2′-deoxyguanosine

## Abstract

It was investigated, using a female rat model of low and moderate exposure of human to cadmium (Cd, 1 and 5 mg Cd/kg diet for 3–24 months), whether a polyphenol-rich 0.1% aqueous extract from *Aronia melanocarpa* L. berries (AE) may prevent Cd-induced lipid peroxidation and oxidative modifications of proteins and deoxyribonucleic acid (DNA) in the liver. For this purpose, markers of lipid peroxidation (lipid peroxides and 8-isoprostane) and oxidative injury of proteins (protein carbonyl groups and 3-nitrotyrosine) and DNA (8-hydroxy-2′-deoxyguanosine) were measured in this organ. The expression of metallothionein 1 (MT1) and metallothionein 2 (MT2) genes was estimated for a better explanation of the possible mechanisms of protective action of AE against Cd hepatotoxicity. The low and moderate treatment with Cd induced lipid peroxidation and oxidatively modified proteins and DNA, as well as enhanced the expression of MT1 and MT2 in the liver, whereas the co-administration of AE completely prevented almost all of these effects. The results allow us to conclude that the consumption of aronia products under exposure to Cd may offer protection against oxidative injury of the main cellular macromolecules in the liver, including especially lipid peroxidation, and in this way prevent damage to this organ.

## 1. Introduction

The health-promoting impact of numerous compounds naturally occurring in plants (i.e., polyphenols, carotenoids, vitamins, and minerals) and dietary products abundant in these compounds in prevention of various diseases and their treatment is well-known and widely reported [1,2,3,4]. However, in recent years a growing attention of the scientific community, especially nutritionists and toxicologists, has been focused on the possibilities of using the beneficial properties of plant-derived biologically active substances, including polyphenols, and products abundant in these compounds in protection against detrimental health outcomes of exposure to the main and most toxic contaminants of dietary products such as cadmium (Cd) [3,4,5,6].

Cd is a common pollutant of the natural environment and food in developing and industrialized countries and it is forecasted that exposure to this toxic heavy metal will grow [4,5,7,8]. Moreover, an increasing amount of evidence shows that even very low exposure to this xenobiotic throughout a lifetime may contribute to the occurrence of various unfavorable health consequences [4,5,9,10]. Therefore, searching for an effective method of preventing adverse health effects of exposure to this metal is one of the main issues the attention of researchers has been focused on. 

Owing to the fact that Cd toxicity is strictly connected with its pro-oxidative action, the main interest in looking for the most effective strategy of prevention from the dangerous effects of its action has been attached to natural antioxidants, especially polyphenolic compounds [4,5,6,11,12,13,14,15]. Polyphenols are the biggest group of biologically active compounds occurring in plant-derived food (fruits, vegetables, and grains), herbs, and drinks (mainly green and black tea and red wine) [3,4,6,14,15]. These compounds, due to their abundance in hydroxyl groups (-OH groups), are able to chelate ions of toxic metals, including Cd ions (Cd^2+^), and inactivate free radicals (FR) [16,17,18]. 

Literature data indicate that some polyphenolic compounds (i.e., anthocyanins, quercetin, and naringenin) or polyphenol-rich products (i.e., aronia berries, blueberry, ginger, and purple carrot) may provide protection against Cd toxicity [4,6,11,12,13,14,15,17,19,20,21,22,23,24]. However, among polyphenol-rich food products the especially promising option in this regard seems to be the berries of *Aronia melanocappa* L. (Aronia melanocarpa (Michx.) Elliott; Rosaceae) (chokeberries) which are characterized by one of the highest contents of these compounds, particularly anthocyanins [3,19]. 

Recently, using a rat model (females) of low-level and moderate human exposure to Cd during a lifetime (1 and 5 mg Cd/kg diet, respectively, for up to 24 months) we have observed that administration of an extract from *A. melanocarpa* berries (AE) resulted in a decrease in the body burden of this xenobiotic (Table S1) [17], and also prevented disturbances in the metabolism and the weakening of the strength properties of bone [12,19,20] and the destruction of the oxidative/antioxidative balance in the liver (Table S2) [11], bone tissue, and serum [12]. The extract also provided protection from pathological changes in the morphological structure of the liver [11] and decreased the concentrations of Cd and metallothionein (MT) and improved the status of zinc (Zn) and copper (Cu) in the main organs of Cd storage (liver and kidneys) [13,17]. Moreover, it has been reported that the administration of aronia anthocyanins to rats exposed to Cd diminished the accumulation of this toxic metal in the liver and kidneys and decreased the activities of enzymatic markers of liver damage in the serum [21].

Liver, due to its important role in the storage and detoxification of Cd, is especially vulnerable to injury caused by this xenobiotic. Recent epidemiological and experimental data show that even low-level, long-term exposure to Cd creates a risk of damage to this organ [4,5,9,10,11]. Cellular and intracellular membranes have been recognized to be the targets for the damaging action of this xenobiotic, and lipid peroxidation has been considered as an important mechanism of its hepatotoxicity. Oxidative damage to cellular macromolecules may result in serious injury of hepatocytes, leading to morphological and functional changes in the liver, which may have detrimental consequences for the organism [4,5,11,25]. Therefore, it is very important to find an effective way to protect against liver injury, especially by chemical substances to which humans are exposed within a lifetime and which, like Cd, progressively accumulate in this organ [4,17], and also important to explain the mechanisms of this protection. 

Taking into account our finding that the administration of AE at the conditions of low and moderate treatment with Cd prevented oxidative stress in the liver (Table S2) [11], we have hypothesized that the extract may also protect against enhanced lipid peroxidation and oxidative modifications of proteins and deoxyribonucleic acid (DNA), and in this way provide protection from this organ damage. To investigate this hypothesis, biomarkers of oxidative injury of the key cellular macromolecules in the liver were assayed. Moreover, to better explain the possible mechanisms of beneficial influence of AE regarding Cd hepatotoxicity, it was necessary to investigate whether the extract may mediate the biosynthesis of MT which is the protein that plays a key role in the accumulation and detoxification of this metal [13,26]. It was also important because FR and reactive oxygen species (ROS) may influence protein biosynthesis at the stage of transcription and translation [26] and we have noted that the extract consumption under the intoxication with Cd decreased the concentration of MT in the liver (Table S3) [13]. Thus, the expression of metallothionein 1 (MT1) and metallothionein 2 (MT2) messenger ribonucleic acid (mRNA) in the liver tissue were evaluated as well. Apart from that, the FR scavenging activity of the extract was measured to estimate its antioxidative potential. This investigation is a part of wide-designed and comprehensive research on the possibility of using AE in prevention from the deleterious health effects of long-term exposure to Cd and the mechanisms of this protection, and it provides new, important, and clinically promising data on this subject.

## 2. Materials and Methods

### 2.1. Chemicals

Dipotassium hydrogen phosphate and potassium dihydrogen phosphate were obtained from POCh (Gliwice, Poland). Morbital and heparin were purchased from Biowet (Pulawy, Poland) and Biochemie GmbH (Kundl, Austria), respectively. Butyl-hydroxytoluene was obtained from Sigma-Aldrich Gmbh (Steinheim, Germany). The kits for the determination of lipid peroxides (LPO), 8-hydroxy-2′-deoxyguanosine (8-OHdG), and Nuclear Extraction Kit used for DNA extraction were obtained from OxisResearch (Foster City, CA, USA). Chemicals for the measurements of 8-isoprostane and protein carbonyl groups (PC) were provided by the Cayman Chemical Company (Ann Arbor, MI, USA). The kit for assay of concentration of 3-nitrotyrosine (3-NT) was purchased from Wuhan EIAab Science Co., Ltd. (Wuhan, China). Protein concentration was evaluated using the BioMaxima kit (Lublin, Poland). Isolation of ribonucleic acid (RNA) was performed with the use of TRIsure^TM^ (Bioline, London, UK), chloroform, isopropyl alcohol, ethyl alcohol (Sigma-Aldrich, Saint Louis, MO, USA), and diethyl dicarbonate (DEPC)-treated water (Bioline). Reverse transcriptase polymerase chain reaction (RT-PCR) and real-time polymerase chain reaction (Real-Time PCR) were performed with SensiFAST^TM^ complementary DNA (cDNA) Synthesis Kit and SensiFAST™ SYBR No-Rox Kit by Bioline, respectively. Ribonuclease-free deoxyribonuclease (RNase-free DNase) was purchased from Bio-Rad (Hercules, CA, USA). Oligonucleotides used in the PCR were synthesized by Genomed (Warsaw, Poland). 1,1-diphenyl-2-picrylhydrazyl radical (DPPH**^·^**) and 6-hydroxy-2,5,7,8-tetramethylchroman-2-carboxylic acid (Trolox) were provided by Sigma-Aldrich. Methanol was purchased from POCh.

### 2.2. Cd Diets

The diets containing Cd (1 and 5 mg Cd/kg) were delivered by Label Food “Morawski’’ (Kcynia, Poland). They were prepared by the addition of cadmium chloride 2.5-hydrate (CdCl_2_ × 2.5 H_2_O; POCh) into the components of the Labofeed H diet (breeding diet, administered during the first 3 months of the experiment) and standard Labofeed B diet (maintenance diet, used since the 4th month of the study up to its end) at the stage of their production. A procedure was performed by us checking the homogeneity of the 1 and 5 mg Cd/kg diets in terms of Cd content, which confirmed that this metal concentration in these diets perfectly agreed with the certified values (1.09 ± 0.13 mg/kg (mean ± standard deviation–SD) and 4.92 ± 0.53 mg/kg, respectively) [17]. Mean Cd concentration quantified by us in the standard Labofeed diets (breeding and maintenance diets) reached 0.0584 ± 0.0049 mg/kg [17].

### 2.3. A. Melanocarpa Extract

A lyophilized powdered chokeberry extract produced by Adamed Consumer Healthcare (Tuszyn, Poland) was used. The extract contained 65.74% of polyphenolic compounds, including 18.65% of anthocyanins (manufacturer’s declaration; Certificate KJ 4/2010). The concentration of total polyphenols determined by us in the powdered extract reached 612.40 ± 3.33 mg/g (mean ± standard error—SE) [19]. The following polyphenolic compounds were quantified in the extract: total anthocyanins (202.28 ± 1.28 mg/g); cyanidin derivatives such as cyanidin 3-O-β-galactoside (80.07 ± 1.05 mg/g), cyanidin 3-O-α-arabinoside (33.21 ± 0.01 mg/g), and cyanidin 3-O-β-glucoside (3.68 ± 0.01 mg/g), total proanthocyanidins (129.87 ± 1.12 mg/g), total phenolic acids (110.92 ± 0.89 mg/g) and chlorogenic acid (68.32 ± 0.08 mg/g), as well as total flavonoids (21.94 ± 0.98 mg/g) [19]. Other components such as carotenoids, pectins, sugar, sugar alcohols (parasorboside and sorbitol), phytosterols, triterpenes, vitamins, and minerals were also present in the extract (producer data,3). According to the manufacturer data, the extract was 6.1% water.

### 2.4. Animals

The investigation was performed on 3–4 weeks old 192 female Wistar rats (Crl: WI (Han)) delivered by certified breeding (Laboratory Animal House in Brwinów, Poland). Before beginning of the experiment, the rats were allowed for acclimatization to the laboratory conditions for 5 days. The animals were maintained in conventional environment controlled throughout the experiment (temperature of 22 ± 2 °C, relative humidity of 50 ± 10%, and 12-h light/dark cycle) with free access to feed and drinking water. The experiment was conducted according to the ethical principles and institutional guidelines as well as International Guide for the Use of Animals in Biomedical Research. The study received approval of the Local Ethics Committee for Animal Experiments in Bialystok (Poland) (approval numbers 60/2009 and 34/2015 on 21 September 2009 and 25 March 2015, respectively). 

### 2.5. Study Design

Animals were randomly allotted into 6 groups, each of which had 32 individuals. One of these groups was administered with 0.1% aqueous solution of AE used as the only drinking fluid (AE group), two groups were intoxicated with Cd alone in the diet at the concentration of 1 or 5 mg Cd/kg (Cd_1_ and Cd_5_ group), while the next two groups received AE during the whole course of the exposure to Cd (Cd_1_ + AE group and Cd_5_ + AE group) for 3, 10, 17, and 24 months. The last group received standard Labofeed diet (0.0584 ± 0.0049 mg Cd/kg) and redistilled water (<0.05 μg Cd/L) and it served as a control. More details about the experimental model have been provided in our previous publications [11,12,13,17,19,20]. 

AE was administered as 0.1% aqueous extract obtained through dissolution of 1 g of lyophilized chokeberry extract by Adamed Consumer Healthcare in 1 L of redistilled water. The concentration of total polyphenols in 0.1% AE amounted to 0.612 ± 0.003 mg/mL (mean ± SE), and was stable during 24 h since the solution preparation, while the concentration of Cd in the extract was <0.05 µg/L [17,19]. The daily intake of AE in the females during the 24-month experiment was in the range of 63.1–159.1 mg/kg body weight (b.w.), and the consumption of polyphenolic compounds reached 41.5–104.6 mg/kg b.w. (the intake of these compounds was higher than their recommended daily intake) and did not differ independently of the manner of the extract application (alone or under the treatment with Cd) [17].

The mean daily intake of Cd in the animals maintained on the 1 and 5 mg Cd/kg diet during the 24-month experimental period, regardless of whether this metal was used alone or co-administered with AE, ranged from 37.50 to 84.88 µg/kg b.w. and from 196.69 to 404.76 µg/kg b.w., respectively [17]. The feeding of the rats with the diet containing 1 mg Cd/kg reflects low lifetime environmental exposure to this xenobiotic, while the higher intoxication (5 mg Cd/kg diet) corresponds to moderate human exposure (Table S1) [17].

After 3, 10, 17, and 24 months of the investigation 8 rats of each group (except for 7 animals in the AE, Cd_1_, and Cd_5_ groups after 24 months) were sacrificed under anesthesia with barbiturate—Morbital (administered intraperitoneally in a dose of 30 mg/kg b.w.). The whole blood was collected by cardiac puncture with or without heparin (as an anticoagulant) and various tissues and organs, including the liver, were anatomized. Biological material that had not been used immediately was preserved frozen at −70 °C until all determinations were performed.

In order to estimate the influence of the consumption of AE on oxidative modifications of the liver macromolecules, markers of lipid peroxidation (LPO and 8-isoprostane) and oxidative damage to proteins (PC and 3-NT) and DNA (8-OHdG) were evaluated. The expression of MT1 and MT2 mRNA in the liver tissue was assayed to determine whether the extract may influence biosynthesis of this protein. The impact of Cd and/or AE on the investigated parameters at particular time points was evaluated in various subgroups within particular experimental groups. Moreover, the measurement of the FR scavenging activity of 0.1% AE was performed as well.

### 2.6. Analytical Procedures

#### 2.6.1. Determination of the Indices of Oxidative Changes in the Liver

##### Preparation of the Liver Tissue Homogenates

In aim to quantify the concentrations of biomarkers of lipid peroxidation and oxidative damage to proteins, 10% (weight/volume—*w*/*v*) homogenates of the liver tissue were made. For this purpose, previously weighted liver slices were subjected to homogenization in 50 mM potassium phosphate buffer (pH = 7.4; received by mixing 1 M dipotassium hydrogen phosphate, 1 M potassium dihydrogen phosphate, and distilled water) with the admixture of butyl-hydroxytoluene (antioxidant) by usage of a high-performance homogenizer (Ultra-Turrax T25, IKA, Staufen, Germany). In this way prepared homogenates were centrifuged (700× *g*) for 20 min at 4 °C with the use of MPW-350R centrifugator (Medical Instruments, Warsaw, Poland) [27]. The aliquots were collected and kept frozen at −70 °C until all parameters were determined. 

In order to estimate the concentration of 8-OHdG, 25% (*w*/*v*) liver homogenates were done by using Dounce homogenizer (Schutt Labortechnik GmbH, Göttingen, Germany). An extraction of genetic material was performed by using the Nuclear Extraction Kit according to the producent’s instruction. The cytoplasmic and nuclear fractions of DNA were extracted with a detergent and inhibitors of protease and phosphatase included in the kit.

##### Determination of the Markers of Lipid Peroxidation and Oxidative Damage to Proteins and DNA

The concentration of LPO (including malondialdehyde—MDA and 4-hydroxyalkenal—HNE) was evaluated by using Bioxytech LPO-586 kit. In the assay a chromogenic reagent—N-methyl-2-phenylindole reacts with MDA and HNE at 45 °C forming a stable chromophore characterized by maximal absorbance at 586 nm. The intra-assay coefficient of variation (CV) was <4.95%. Assay of 8-isoprostane was made by using the 8-isoprostane Enzyme Immunoassay (EIA) Kit with the intra- and inter-assay CV <5% and <2%, respectively. The liver concentration of 3-NT was measured with the use of the General Nitrotyrosine Enzyme-Linked Immunosorbent Assay (ELISA) Kit. The intra-assay CV was <3.75% and the inter-assay CV was <2.5%. PC concentration in the liver homogenates was measured spectrophotometrically, based on the reaction of PC with 2,4-dinitrophenylhydrazine, with the use of Protein Carbonyl Assay Kit (intra-assay CV <2%). The concentration of 8-OHdG was assayed with the use of Bioxytech 8-OHdG EIA Diagnostic Kit with the intra-assay CV <3.4% and inter-assay CV <6%.

All the indices of the oxidative changes in the liver were adjusted for the concentration of protein, which was quantified with the use of the Total Protein BioMaxima Kit (intra-assay CV <2.2%). 

#### 2.6.2. RNA Isolation and Real-Time PCR for Estimation of the Expression of MT1 and MT2 mRNA in the Liver

Liver samples (50 mg) were subjected to homogenization in 1 mL of TRIsure^TM^ reagent. Next, total RNA was extracted with chloroform to remove proteins and genomic DNA and precipitated with cold isopropyl alcohol. The RNA precipitate was washed with 75% (volume/volume—*v*/*v*) ethyl alcohol and dissolved in DEPC-treated water. In addition, RNA extracts were treated with RNase-free DNase Ι to be sure that RNA was completely purified from DNA. The concentration and quality of isolated RNA were quantified on a spectrophotometer (Nanodrop 2000, Thermo Scientific, Waltham, MA, USA) and until the measurements of gene expression the purified RNA was stored at −70 °C.

1 μg of total RNA was reverse-transcribed into cDNA with SensiFAST^TM^ cDNA Synthesis Kit in reaction mixtures containing a unique blend of random hexamer and anchored oligo dT primers, reverse transcriptase and TransAmp Buffer in a final volume of 20 μL. Reaction mixtures were sequentially incubated at 25 °C for 10 min, 42 °C for 15 min, and at 85 °C for 5 min.

Real-time quantitative PCR was conducted in a CFX96 Real-Time System (Bio-Rad). 2 μL of 1:3 diluted with DEPC-treated water cDNA sample from the RT-PCR reaction was added to 18 μL of master mix containing 0.8 μL of each (forward and reverse) 10 μM primer (to a final concentration of 400 nM), 10 μL of SensiFAST™ SYBR No-Rox Mix, and 6.4 μL of DEPC-treated water. Primer sequences (forward and reverse) used to amplify the cDNA of the studied genes and the size of the products were as follows: MT1 forward 5′-GCG TCA CCA CGA CTT CAA C-3′ and MT1 reverse 5′-GTC ACA TCA GGC ACA GCA C-3′ (266 base pair—bp); MT2 forward 5′-AAC TGC CGC CTC CAT TCG C-3′ and MT2 reverse 5′-AGC ACT TCG CAC AGC CCA C-3′ (153 bp); glyceraldehyde-3-phosphate dehydrogenase (GAPDH) forward 5′-CCT TCA TTG ACC TTC ACT ACA TGG TCT A-3′ and GAPDH reverse 5′-TGG AAG ATG GTG ATG GCC TTT CCA TTG-3’ (126 bp) [28]. The thermal cycling conditions included 40 cycles of denaturation at 95 °C for 10 s, annealing at 60 °C for 10 s, and extension at 72 °C for 15 s. A melt curve was subsequently performed with a melting protocol from 55 °C to 95 °C with a 0.2 °C increment and 1 s holding at each increment to analyze the generated products. The relative expression of the MT1 and MT2 genes normalized to GAPDH was estimated based on the delta–delta Ct method [29]. 

#### 2.6.3. Evaluation of the FR Scavenging Activity of 0.1% AE

FR scavenging activities of the 0.1% extract and Trolox (used as a standard) were determined with the DPPH**^·^** assay based on the ability of antioxidants present in an investigated sample to reduce the stable dark violet DPPH**^·^** to the yellow colored diphenyl-picrylhydrazine [30]. Briefly, 100 μL of the methanol solution of DPPH**^·^** at the concentration of 0.2 mg/mL were added to 100 μL of 0.1% AE and standard (Trolox at the concentration of 0.25 mg/mL). After 30 min of incubation at room temperature, the absorbance of the solutions was recorded at 517 nm (SPECORD 40, Analytik Jena, Jena, Germany). The activity of each sample was determined by comparing its absorbance with that of a control solution (reagents without the extract) and the standard. The capability to scavenge DPPH**^·^** was calculated using the following formula: DPPH**^·^**scavenging effect (%) = [(X_control_ − X_extract_/X_control_) × 100], where X_control_ is the absorbance of the control sample and X_extract_ is the absorbance in the presence of the extract [30]. The assays were performed in triplicate. The assay precision, reflected as the intra-assay CV, was <0.44%.

### 2.7. Statistical Analysis

**Figure 1:**
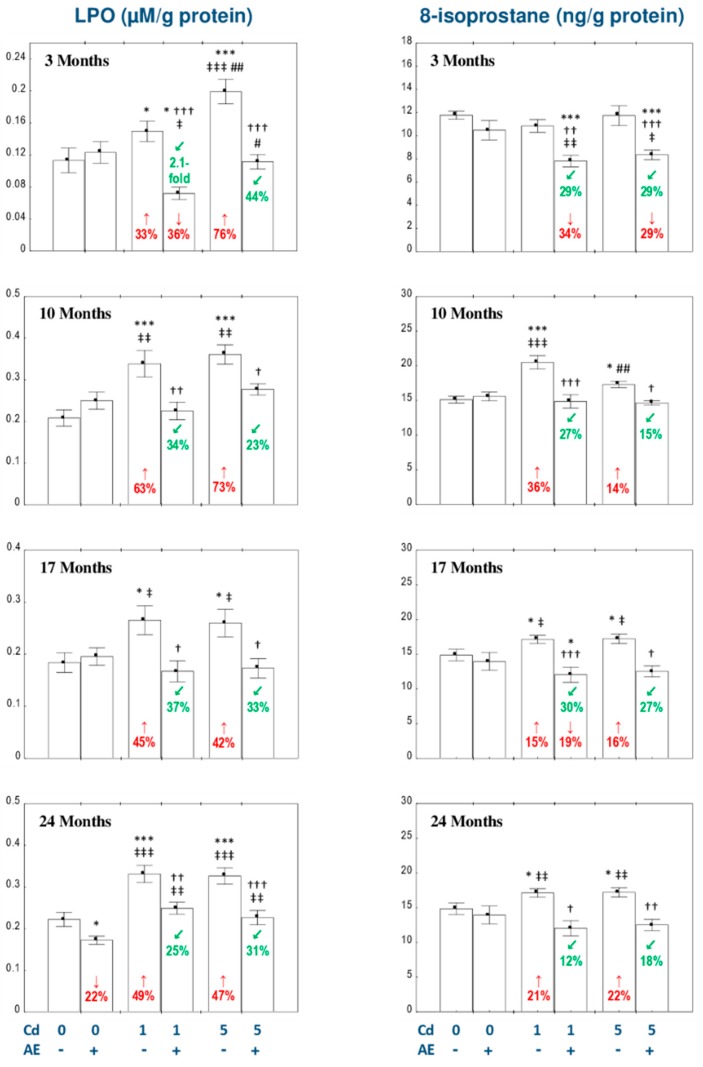
The influence of the extract from *Aronia melanocarpa* L. berries (AE) on the concentrations of lipid peroxides (LPO) and 8-isoprostane in the liver of female rats exposed to cadmium (Cd). The females were given Cd in the diet at the concentration of 0, 1, and 5 mg Cd/kg and/or 0.1% aqueous AE (+) or not (−). Data are represented as mean ± SE for 8 animals, except for 7 females in the AE, Cd_1_, and Cd_5_ groups after 24 months. Statistically significant differences (ANOVA, Duncan’s multiple range post hoc test): * p < 0.05, *** *p* < 0.001 vs. control group; ^†^ *p* < 0.05, ^††^ *p* < 0.01, ^†††^ *p* < 0.001 vs. appropriate group treated with Cd alone; ^‡^ *p* < 0.05, ^‡‡^ *p* < 0.01, ^‡‡‡^ *p* < 0.001 vs. group administered with AE alone; ^#^ *p* < 0.05, ^##^ *p* < 0.01 vs. appropriate group receiving the 1 mg Cd/kg diet (alone or with AE). Numerical values in bars or above the bars disclose the percentage changes or factors of changes in comparison to the control group (↑, increase; ↓, decrease) or the appropriate group given Cd alone (↙, decrease).

**Figure 2:**
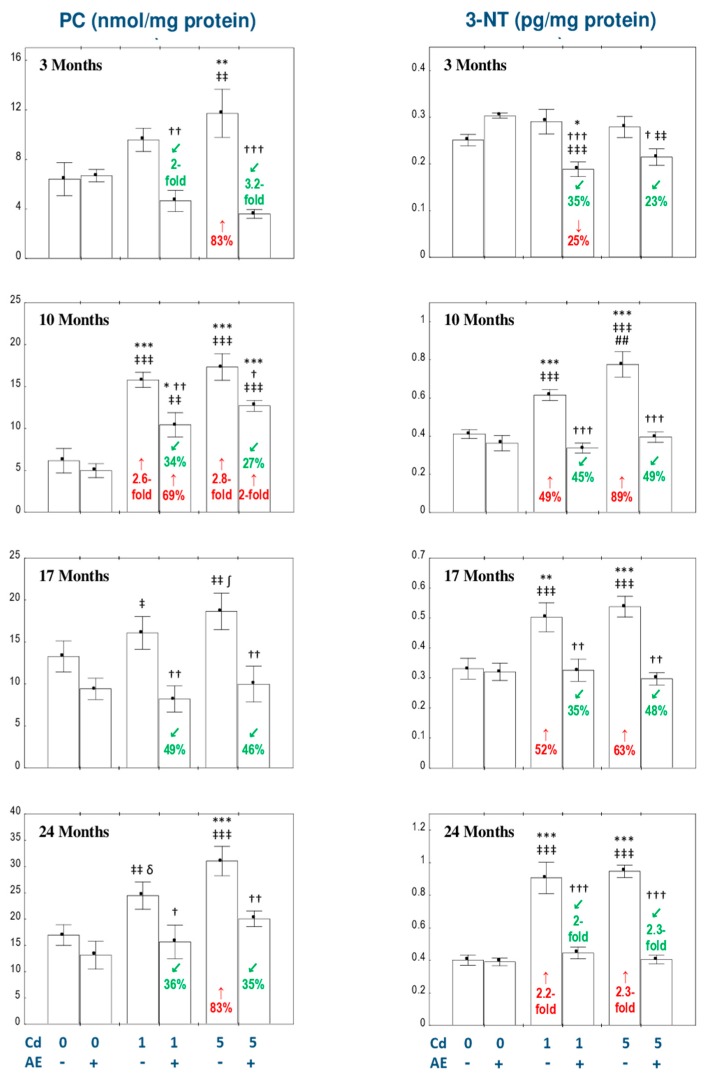
The influence of the extract from *Aronia melanocarpa* L. berries (AE) on the concentrations of protein carbonyl groups (PC) and 3-nitrotyrosine (3-NT) in the liver of female rats exposed to cadmium (Cd). The females were given Cd in the diet at the concentration of 0, 1, and 5 mg Cd/kg and/or 0.1% aqueous AE (+) or not (-). Data are represented as mean ± SE for 8 animals, except for 7 females in the AE, Cd_1_, and Cd_5_ groups after 24 months. Statistically significant differences (ANOVA, Duncan’s multiple range post hoc test): * p < 0.05, ** *p* < 0.01, *** *p* < 0.001, ^δ^ *p* = 0.05, ^∫^ *p* = 0.06 vs. control group; ^†^ *p* < 0.05, ^††^ *p* < 0.01, ^†††^ *p* < 0.001 vs. appropriate group treated with Cd alone; ^‡^ *p* < 0.05, ^‡‡^ *p* < 0.01, ^‡‡‡^ *p* < 0.001 vs. group administered with AE alone; ^##^ *p* < 0.01 vs. appropriate group receiving the 1 mg Cd/kg diet alone. Numerical values in bars or above the bars disclose the percentage changes or factors of changes in comparison to the control group (↑, increase; ↓, decrease) or the appropriate group given Cd alone (↙, decrease).

**Figure 3:**
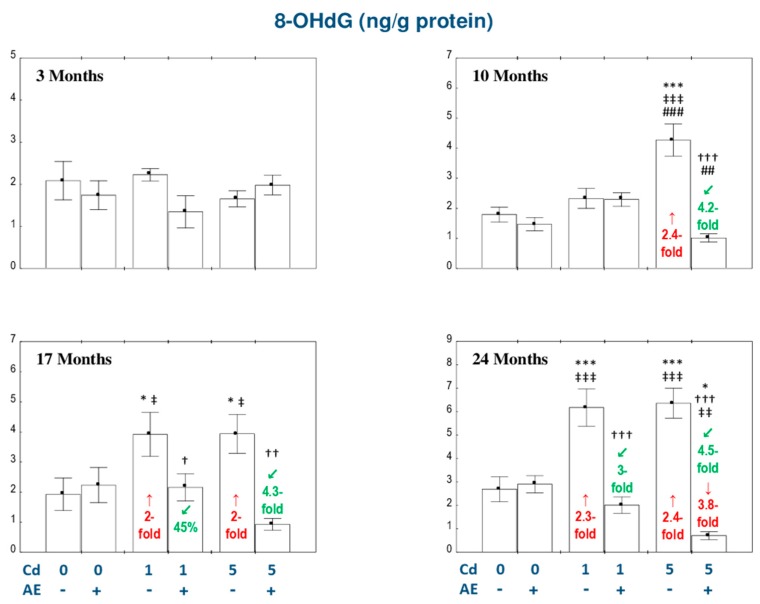
The influence of the extract from *Aronia melanocarpa* L. berries (AE) on the concentration of 8-hydroxy-2′-deoxyguanosine (8-OHdG) in the liver of female rats exposed to cadmium (Cd). The females were given Cd in the diet at the concentration of 0, 1, and 5 mg Cd/kg and/or 0.1% aqueous AE (+) or not (-). Data are represented as mean ± SE for 8 animals, except for 7 females in the AE, Cd_1_, and Cd_5_ groups after 24 months. Statistically significant differences (ANOVA, Duncan’s multiple range post hoc test): * p < 0.05, *** *p* < 0.001 vs. control group; ^†^ *p* < 0.05, ^††^ *p* < 0.01, ^†††^ *p* < 0.001 vs. appropriate group treated with Cd alone; ^‡^ *p* < 0.05, ^‡‡^ *p* < 0.01, ^‡‡‡^ *p* < 0.001 vs. group administered with AE alone; ^##^ *p* < 0.01, ^###^ *p* < 0.001 vs. appropriate group receiving the 1 mg Cd/kg diet (alone or with AE). Numerical values in bars or above the bars disclose the percentage changes or factors of changes in comparison to the control group (↑, increase; ↓, decrease) or the appropriate group given Cd alone (↙, decrease).

**Figure 4:**
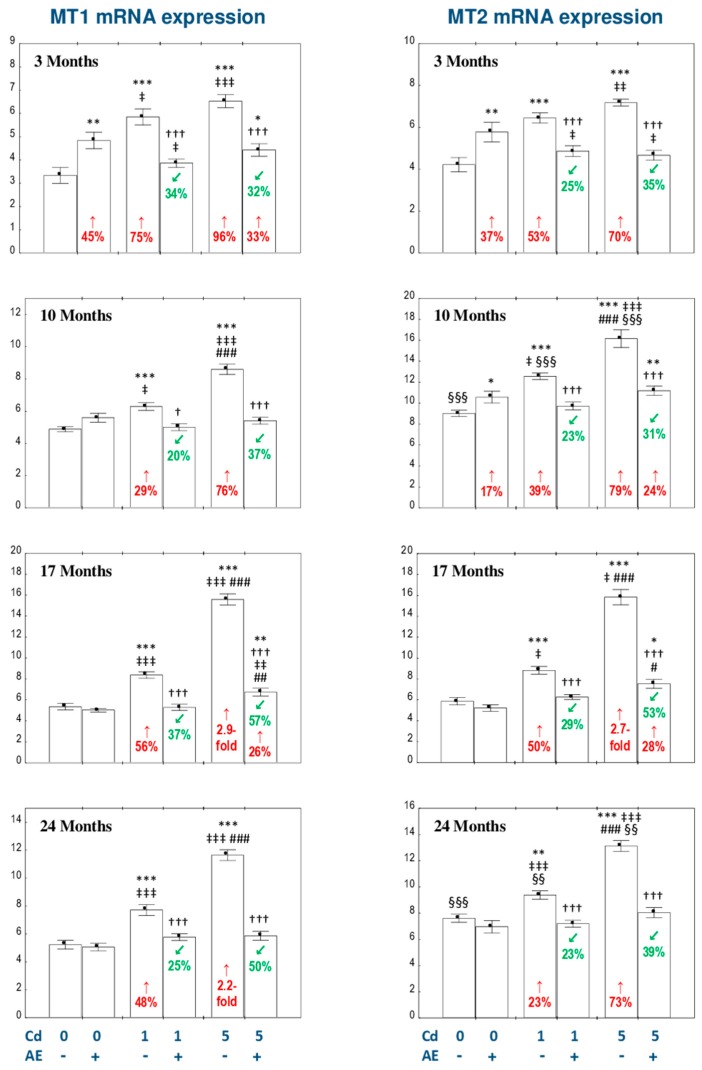
The influence of the extract from *Aronia melanocarpa* L. berries (AE) on the expression of metallothionein 1 (MT1) and metallothionein 2 (MT2) messenger ribonucleic acid (mRNA) in the liver of female rats exposed to cadmium (Cd). The females were given Cd in the diet at the concentration of 0, 1, and 5 mg Cd/kg and/or 0.1% aqueous AE (+) or not (−). Data are represented as mean ± SE for 8 animals, except for 7 females in the AE, Cd_1_, and Cd_5_ groups after 24 months. Statistically significant differences (ANOVA, Duncan’s multiple range post hoc test): * p < 0.05, ** *p* < 0.01, *** *p* < 0.001 vs. control group; ^†^ *p* < 0.05, ^†††^ *p* < 0.001 vs. appropriate group treated with Cd alone; ^‡^ *p* < 0.05, ^‡‡^ *p* < 0.01, ^‡‡‡^ *p* < 0.001 vs. group administered with AE alone; ^#^ *p* < 0.05, ^##^ *p* < 0.01, ^###^ *p* < 0.001 vs. appropriate group receiving the 1 mg Cd/kg diet (alone or with AE); ^§§^ *p* < 0.01, ^§§§^ *p* < 0.001 vs. MT1 in appropriate experimental group. Numerical values in bars disclose the percentage changes or factors of changes in comparison to the control group (↑, increase) or the appropriate group given Cd alone (↙, decrease).

Statistical analysis of detailed results was performed with the usage of statistical software Statistica 12 (StatSoft, Tulsa, OK, USA). Data for 8 rats in each group (except for 7 animals in the AE, Cd_1_, and Cd_5_ groups after 24 months) are presented as a mean ± SE. A one-way analysis of variance (ANOVA) was conducted to discern if there were statistically significant differences among experimental groups (level of statistical significance—*p* < 0.05). The Duncan’s multiple range post hoc test was used to compare individual groups and determine which two means differed statistically significantly (*p* < 0.05). In figures (Figure 1, Figure 2, Figure 3 and Figure 4), statistically significant differences in comparison to the control group, the appropriate group treated with Cd alone (Cd_1_ + AE vs. Cd_1_ and Cd_5_ + AE vs. Cd_5_), the group administered with AE alone (Cd_1_ vs. AE, Cd_1_ + AE vs. AE, Cd_5_ vs. AE, Cd_5_ + AE vs. AE), and the appropriate group maintained on the 1 mg Cd/kg diet alone or with AE (Cd_5_ vs. Cd_1_ and Cd_5_ + AE vs. Cd_1_ + AE) at each time point are marked. Moreover, in Figure 4 statistically significant differences between the expression of MT1 and MT2 in the appropriate experimental groups are indicated. A two-way analysis of variance (ANOVA/MANOVA, test *F*) was performed in order to disclose the possible interactions between Cd and AE and their main effects (test *F*, *p* < 0.05). In the case when the ANOVA/MANOVA analysis showed an interactive effect of Cd and AE, additional calculations were conducted to estimate the possible character of the interaction. The effect of co-administration of Cd and AE was compared to the sum of effects of their separate administration. The effect of Cd or/and AE was expressed as a percentage of change or a factor of change of a measured parameter compared to the control group. Based on the obtained results it was estimated whether the interaction had an antagonistic (Cd + AE effect < Cd effect + AE effect), additive (Cd + AE effect = Cd effect + AE effect) or other character [31]. The Spearman rank correlation analysis between the measured indices of oxidative injury of the liver macromolecules and the concentrations of Cd and hydrogen peroxide (H_2_O_2_), total antioxidative status (TAS), total oxidative status (TOS), and the level of oxidative stress expressed as oxidative stress index (OSI) in this organ presented in our previous reports from studies in this experimental model [11,17] was performed as well. Furthermore, the correlations between MT1 and MT2 mRNA expression, as well as the expression of these MTs and the previously determined liver concentrations of Cd and MT [13,17] were investigated. Correlations between variables were recognized as statistically significant at the values of correlation coefficients (*r*) having *p* < 0.05. 

## 3. Results 

### 3.1. The Influence of AE on Lipid Peroxidation in the Liver of Female Rats Exposed to Cd

AE alone had no influence on the concentrations of LPO and 8-isoprostane in the liver, except for a decrease in LPO concentration after 24 months of the extract administration (Figure 1). 

The low-level and moderate treatment with Cd resulted in an increase in the concentrations of LPO at each time point (by 33–76%) and 8-isoprostane after 10–24 months (by 14–36%) of the study, while the simultaneous administration of AE entirely prevented these effects of this xenobiotic (Figure 1). Furthermore, in the Cd_1_ + AE group the concentration of LPO after 3 months and the concentration of 8-isoprostane after 17 months were even lower compared to the control animals (Figure 1). The consumption of AE during the 3-month treatment with the 1 and 5 mg Cd/kg diet decreased the unchanged by Cd alone concentration of 8-isoprostane compared to both control group and the respective Cd group (Figure 1). 

Generally, there were no differences between the indices of lipid peroxidation in the liver dependent on the level of exposure to Cd (Figure 1). However, in the Cd_5_ and Cd_5_ + AE groups, the concentration of LPO after 3 months was higher than in the Cd_1_ and Cd_1_ + AE groups (by 33% and 56%, respectively; Figure 1), while the concentration of 8-isoprostane in the Cd_5_ group after 10 months was lower (by 16%) than in the Cd_1_ group (Figure 1). 

The ANOVA/MANOVA analysis revealed that the beneficial influence of AE administration during the exposure to Cd on the concentrations of LPO and 8-isoprostane resulted from independent action of the extract ingredients (*F* = 4.326–20.51, *p* < 0.05–0.001) and/or their interaction with this toxic element (*F* = 4.277–14.74, *p* < 0.05–0.001; Table 1). The interactive impact of Cd and AE on the concentrations of the measured indices of lipid peroxidation seemed to be antagonistic in character (Table 1). However, the two-way analysis of variance revealed the lack of a statistically significant independent impact of AE and its interaction with Cd (Table 1) on the concentration of 8-isoprostane in the Cd_1_ + AE group after 24 months, in spite of the clear protective impact of the extract consumption under Cd exposure evaluated on the basis of the findings of one-way analysis of variance (Figure 1).

The liver concentrations of LPO and 8-isoprostane positively correlated with the concentration of Cd in this organ (*r* = 0.332, *p* < 0.001 and *r* = 0.172, *p* < 0.05, respectively). Moreover, positive correlations occurred between the concentrations of LPO and 8-isoprostane and the value of TOS (*r* = 0.227, *p* < 0.01 and *r* = 0.197, *p* < 0.01, respectively) and the concentration of H_2_O_2_ (*r* = 0.756, *p* < 0.001 and *r* = 0.751, *p* < 0.01, respectively) in this organ.

### 3.2. The Influence of AE on Oxidative Protein Damage in the Liver of Female Rats Exposed to Cd

The consumption of AE alone during the whole experimental period had no effect on the concentrations of PC and 3-NT in the liver (Figure 2). 

The low-level exposure to Cd increased (2.6-fold) the concentration of PC after 10 months, whereas the moderate intoxication with this toxic metal increased (by 83% to 2.8-fold) the value of this biomarker of oxidative modifications of proteins after 3, 10, and 24 months of the experiment (Figure 2). Moreover, the concentration of PC in the animals fed with the 1 mg Cd/kg diet for 24 months was on the border of statistical significance (*p* = 0.05), while in the ones maintained on the diet containing 5 mg Cd/kg for 17 months it clearly tended to be higher (*p* = 0.06) than in the control group (the mean values were higher by 44% and 41% compared to the control, respectively; Figure 2). The administration of AE in the conditions of the low-level and moderate intoxication with Cd decreased the level of this parameter at each time point and completely prevented all changes caused by this metal, except for the Cd_1_ + AE and Cd_5_ + AE groups after 10 months of the experiment, in which the protective effect was only partial (Figure 2). 

In the females given the diet containing 1 and 5 mg Cd/kg, the concentration of 3-NT was elevated (from 49% up to 2.3-fold) after 10, 17, and 24 months, whereas the concomitant application of the extract offered total protection from these changes (Figure 2). Moreover, the consumption of AE during the 3-month exposure to both Cd levels diminished the concentration of 3-NT compared to the respective group intoxicated with this metal alone and towards the control group at the low-level treatment (Figure 2).

No differences in the liver concentrations of PC and 3-NT were observed between the appropriate groups receiving the 1 and 5 mg Cd/kg diet alone or with AE (Cd_1_ vs. Cd_5_ and Cd_1_ + AE vs. Cd_5_ + AE), except for a higher (by 26%) concentration of 3-NT in the Cd_5_ group than in the Cd_1_ group after 10 months (Figure 2).

The two-way analysis of variance disclosed that the effect of the chokeberry extract on the concentration of PC and 3-NT in the liver was caused by independent influence of its ingredients (*F* = 5.675–82.46, *p* < 0.05–0.001) and/or their interactive action with Cd (*F* = 4.872–75.97, *p* < 0.05–0.001; Table 2). The interactive impact of Cd and AE on the determined indices of oxidative protein damage had antagonistic character (Table 2).

The concentration of PC and 3-NT positively correlated with the concentration of Cd (*r* = 0.357, *p* < 0.001 and *r* = 0.282, *p* < 0.001, respectively) in the liver. Positive correlations were also disclosed between PC concentration and TOS (*r* = 0.158, *p* < 0.05) and H_2_O_2_ concentration (*r* = 0.574, *p* < 0.001), as well as between 3-NT concentration and TOS (*r* = 0.197, *p* < 0.01), H_2_O_2_ concentration (*r* = 0.845, *p* < 0.001), and the value of OSI (*r* = 0.171, *p* < 0.05).

### 3.3. The Influence of AE on Oxidative DNA Damage in the Liver of Female Rats Exposed to Cd

The consumption of AE alone had no effect on 8-OHdG concentration in the liver throughout the 24-month experimental period (Figure 3). 

The intoxication with the diet containing 1 mg Cd/kg increased the concentration of 8-OHdG after 17 and 24 months (2- and 2.3-fold, respectively), while the moderate exposure to this metal increased (from 2- to 2.4-fold) the concentration of this biomarker already from the 10th month of the investigation (Figure 3). The co-administration of AE completely prevented this heavy metal-caused growth in the concentration of 8-OHdG (Figure 3). Apart from that, the value of this parameter in the Cd_5_ + AE group after 24 months was lower (as much as 3.8-fold) than in the control animals (Figure 3). 

The only differences in the concentration of 8-OHdG between the particular groups given the 1 and 5 mg Cd/kg diet alone or together with AE (Cd_1_ vs. Cd_5_ and Cd_1_ + AE vs. Cd_5_ + AE) were higher (by 83%) value of this biomarker in the Cd_5_ group compared to the Cd_1_ group and lower (by 56%) value of this parameter in the Cd_5_ + AE group than in the Cd_1_ + AE group after 10 months (Figure 3). 

The beneficial impact of the chokeberry extract on the concentration of 8-OHdG in the liver tissue of the female rats treated with Cd was the result of independent action of its ingredients (*F* = 6.709–35.73, *p* < 0.05–0.001) and their antagonistic interaction with this xenobiotic (*F* = 10.07–41.47, *p* < 0.01–0.001; Table 3). Nevertheless, the ANOVA/MANOVA analysis disclosed the lack of a statistically significant independent impact of the extract and the interaction of its components with Cd (Table 3) on the concentration of 8-OHdG in the Cd_1_ + AE group after 17 months in spite of the entire protection given by AE consumption under exposure to this toxic metal disclosed by ANOVA analysis (Figure 3). 

There was no correlation between the concentrations of 8-OHdG and Cd in the liver. However, a positive dependence was revealed between the concentration of this biomarker of DNA damage and the concentration of H_2_O_2_ (*r* = 0.455, *p* < 0.001) and the value of OSI (*r* = 0.256, *p* < 0.001), and a negative relationship appeared between 8-OHdG concentration and TAS (*r* = −0.258, *p* < 0.001).

### 3.4. The Influence of AE on the Expression of MT1 and MT2 mRNA in the Liver of Female Rats Exposed to Cd

In the animals administered with AE alone an increase in the expression of MT1 mRNA was observed after 3 months of the investigation, while the expression of MT2 was enhanced after 3 and 10 months (Figure 4). 

The low-level and moderate treatment with Cd resulted in a significant increase (from 23% to 2.9-fold) in MT1 and MT2 transcript levels at each time point, while the simultaneous administration of AE decreased the expression of both isoforms of MT (often to the values noted in the control animals; Figure 4).

The expression of both MTs genes was higher in the Cd_5_ group compared to the Cd_1_ group after intoxication longer than 3 months (Figure 4). The only differences in these parameters between the Cd_1_ + AE group and Cd_5_ + AE group were their higher expressions in the animals administered with the extract and the 5 mg Cd/kg diet after 17 months of the study (Figure 4). Moreover, the expression of MT2 was equal to or higher than the expression of MT1 in the respective experimental groups (Figure 4). 

The ANOVA/MANOVA analysis showed that the influence of AE administration to the animals treated with Cd on the expression of MT1 and MT2 mRNA was caused by independent action of ingredients of the extract (*F* = 9.010–168.0, *p* < 0.01–0.001) and/or their interaction with this toxic element, which was antagonistic in character (*F* = 4.805–145.1, *p* < 0.05–0.001; Table 4). 

The expression of MT1 mRNA in the liver positively correlated with the expression of MT2 (*r* = 0.656, *p* < 0.001). Statistically significant positive dependences were noted between the expression of genes of MT1 and MT2 and the concentrations of MT (*r* = 0.567, *p* < 0.001 and *r* = 0.328, *p* < 0.001, respectively) and Cd (*r* = 0.528, *p* < 0.001 and *r* = 0.449, *p* < 0.001, respectively) in this organ.

### 3.5. FR Scavenging Activity of 0.1% AE 

The DPPH**^·^** scavenging activities of 0.1% AE and Trolox (used as the standard) were 75.01 ± 0.11% and 77.44 ± 0.34%, respectively.

## 4. Discussion 

The present investigation is the first one indicating the protective effect of AE against oxidative modifications of the main macromolecules such as lipids, proteins, and DNA caused by Cd in the liver. The research not only provided further evidence for the protective role of the extract regarding the toxic action of this xenobiotic, but it also importantly contributed to an explanation of the possible mechanisms of this protection. Although the research was concentrated first of all on the hepatoprotective effect of AE it also provided important data on the unfavorable impact of low and moderate intoxication with Cd on the liver. This research reveals that this heavy metal may oxidatively damage the key cellular macromolecules in the liver at its low concentration in this organ (0.1447 ± 0.0093 μg/g), blood (0.1884 ± 0.0100 μg/L), and urine (0.2184 ± 0.0081 μg/g of creatinine) [17], corresponding to this toxic element concentrations reported in the general population [5,9,10], and confirms our latest finding [11] that even low-level repeated exposure to this xenobiotic may create a risk of liver injury.

Taking into account our previous results that the feeding of female rats with the diet containing 1 and 5 mg Cd/kg destroyed the oxidative/antioxidative balance and induced oxidative stress in the liver tissue and pathological changes in its morphology [11], it was expected that the exposure will also cause oxidative modifications of the basic cellular macromolecules as it was disclosed in the present investigation. The mechanism of pro-oxidative Cd action is well-known and widely reported (for review see [4]). Although this metal is not a redox-active agent and thus is unable to generate FR and ROS directly, it contributes to the occurrence of oxidative stress via an indirect action by weakening the non-enzymatic and enzymatic antoxidative defense, inducing the activities of oxidases, and increasing the concentrations of pro-oxidants, as well as causing mitochondria injury. In this way it damages cellular macromolecules, membranes, and organelles, influences genes expression, and inhibits the repairing mechanism of DNA [4,11,22,23,24,32]. 

A very significant finding of the present study is disclosing that the enhancement of lipid oxidation, recognized on the basis of LPO concentration, which occurred already due to the shortest of the studied periods of the low exposure, preceded oxidative modifications of proteins and DNA. At the time point at which enhanced lipid peroxidation was noted for the first time, structural changes in the liver morphological appearance (lobes with blurred trabecular structure, vacuolization, enlarged dimensions of cells, and mononuclear cell infiltrations) were reported by us as well [11]. Our findings indicate that cellular and intracellular membranes, which, due to lipids abundance are especially vulnerable to oxidation, are target sites for Cd action. Moreover, the findings of the present investigation together with our previous results on Cd hepatotoxicity [11] show that the concentration of LPO seems to be a better parameter to detect enhanced oxidative modifications of lipids in the liver caused by low-level exposure to this metal than the concentrations of MDA and 8-isoprostane. Although lipids seem to be the target macromolecules for this xenobiotic, the low-level exposure was also able to cause oxidative modifications of proteins and DNA and thereby to contribute to the damage to the liver recently reported by us in this experimental model [11]. The reactive end products of oxidative lipid modifications, such as MDA and HNE, may enhance the progression of lipid peroxidation and interact with proteins and nucleic acids [33]. The carbonylation and nitration of amino acids in proteins may lead to their partial or total inactivation [34], whereas the excessive generation of 8-OHdG in the liver is a risk factor for cancer [35]. The results of the present study show that DNA seems to be the most resistant molecule to Cd-mediated oxidative alteration; however, it may also be damaged at low exposure. The main ROS responsible for the formation of 8-OHdG is the very reactive hydroxyl radical (HO·) [36]. Cd may induce the formation of HO· indirectly through the displacement of ions of transitive biometals such as iron (Fe) and Cu (i.e., Fe^2^+ and Cu+) from their binding sites, in this way enabling the creation of this radical in the Fenton reaction [4]. Moreover, this metal may inactivate enzymes required for maintaining the integrity of DNA by replacing the essential divalent metals, such as Zn, in their active centers [37]. 

Taking into account the findings of the current investigation and our recent results [11] showing that repeated, even low-level, treatment with Cd may have very serious consequences in the form of oxidative modifications of lipids, proteins, and DNA, and pathological structural changes in the liver, the findings of the study are important and have practical usefulness because they show that the consumption of AE prevented the hepatotoxic action of this xenobiotic. Moreover, it is valid to emphasize that the protection provided by the extract regarding oxidative modifications of the basic cellular macromolecules was almost entire. Detailed evaluation of the results of the ANOVA/MANOVA analysis allows us to recognize that the favorable influence of AE intake during exposure to the 1 and 5 mg Cd/kg diet regarding this xenobiotic-caused lipid peroxidation and oxidative modifications of proteins and DNA resulted from an independent impact of the extract ingredients and their interactive action with this toxic metal. The independent effect of the extract may be explained first of all by its strong antioxidative properties [3,11,12], while the interactive action involves the influence on the body status of Cd (Table S1) [17,18]. Because the exposure to Cd alone increased the concentrations of LPO, 8-isoprostane, PC, 3-NT, and 8-OHdG in the liver, while the co-administration of AE during the treatment with this heavy metal decreased the values of these parameters (in most cases to the values of the control group and sometimes even lower than in the control animals) and the effects of simultaneous administration of Cd and AE were lower than the sum of the effects of their separate action (Cd + AE effect < Cd effect + AE effect), the Cd—AE interactions have been recognized as antagonistic. The antagonistic character of the interactive impact of AE and Cd on the determined indices of oxidative modifications of lipids, proteins, and DNA in the liver may be elucidated by an ability of the extract ingredients to counteract pro-oxidative action of this heavy metal via decreasing its concentration in this organ [17]. 

The fact that the concentration of LPO after 3 months and the concentration of 8-isoprostane after 17 months in the Cd_1_ + AE group, as well as the concentration of 8-OHdG after 24 months in the Cd_5_ + AE group were even lower than in the control group may be explained by stimulation of antioxidative defense by the co-administration of AE possessing strong antioxidative potential. Indeed, recently we have observed that the liver concentrations of pro-oxidants such as H_2_O_2_, myeloperoxidase, and xanthine oxidase after 3 months, as well as the concentration of xanthine oxidase and TOS after 17 months of exposure to the 1 mg Cd/kg diet were lower compared to the control group [11]. In the Cd_5_ + AE group after 24 months of the study the activities of antioxidative agents such as superoxide dismutase and glutathione reductase, as well as the concentration of glutathione S-transferase, total thiol groups, the value of TAS, and the ratio of reduced/oxidized glutathione were higher compared to the control group, while the concentration of oxidized glutathione was lower compared to the control animals [11]. Moreover, we have previously reported [12] that the consumption of AE not only entirely prevented a Cd-induced increase in the bone concentrations of LPO after 3 months and 8-isoprostanes after 17 months of exposure to the 1 mg Cd/kg diet, as well as 8-OHdG after 24 months of intoxication with the diet containing 5 mg Cd/kg, but also made the values of these parameters lower than in the control group. It is worth mentioning that administration of substances characterized by antioxidative properties in the state of balance between antioxidants and pro-oxidants may only have a slight effect, if any, on the level of oxidative modifications of basic cellular macromolecules. In our previous study in this experimental model [11] we have noted that the administration of AE (in spite of its high antioxidative potential) under physiological conditions (without exposure to Cd) had only minor impact on the oxidative/antioxidative balance, but in the case of exposure to this toxic metal it significantly improved the enzymatic and nonenzymatic antioxidative barrier, decreased the concentrations of pro-oxidants, and provided protection from the development of oxidative stress in the liver.

The findings of our recent study [11] together with the capability of AE to scavenge DPPH**^·^** that was determined in the present paper confirm that the ability of the extract to protect against the unfavorable consequences of the action of Cd as a pro-oxidant at low-level and moderate intoxication, resulting in oxidative modifications of basic cellular macromolecules, was caused by its antioxidative properties. The fact that 0.1% AE showed nearly the same DPPH**^·^** scavenging activity as Trolox, a water-soluble derivative of vitamin E recognized as a powerful FR scavenger [38,39], indicates that the extract is characterized by strong antioxidative potential. The high antioxidative capability of AE is determined by an abundance of bioactive constituents such as vitamins, minerals, carotenoids, and especially polyphenolic compounds (anthocyanins, proanthocyanidins, flavonols, and phenolic acids) [3,11,12]. Owing to the occurrence of -OH groups, polyphenols may serve as hydrogen donors that are able to terminate radical chain reactions, including lipid peroxidation, and regenerate non-enzymatic antioxidants [6]. Since the consumption of AE during the treatment with Cd improved the oxidative/antioxidative balance and counteracted the development of oxidative stress in the liver [11], it also prevented oxidative modifications of cellular macromolecules. Correlations revealed between the concentrations of markers of lipids, proteins, and DNA oxidation and previously determined by us in these animals markers of the oxidative/antioxidative status (TOS, TAS, OSI, and H_2_O_2_ concentration) confirm the close relationship between the intensity of oxidative stress and the extent of oxidative modifications of the basic cellular macromolecules. 

It seems that the mechanism of the interactive hepatoprotective impact of AE during the treatment with Cd might result mainly from the ability of the extract ingredients (first of all polyphenols, but also pectin and fiber) to chelate Cd^2+^ and in this way influence the body turnover of this xenobiotic, including its storage in the liver [16,17,18,40,41]. We have already revealed that the administration of AE decreased intestinal absorption and enhanced urinary elimination of this heavy metal in this way decreasing the body burden of Cd, including its retention in the liver (Table S1) [17]. Although, the administration of the extract to the animals maintained on the diet containing 1 mg Cd/kg had only a very slight preventive effect against the liver accumulation of this xenobiotic, its application under the intoxication with the 5 mg Cd/kg diet importantly decreased Cd content in this organ [17]. The fact that the administration of the chokeberry extract throughout the exposure to the 1 mg Cd/kg diet provided complete protection from lipid peroxidation and DNA oxidation and almost completely prevented oxidative proteins modification in spite of only slightly decreased Cd concentration in the liver allows us to recognize that the favorable effect of the extract at low exposure to this heavy metal might be determined first of all by its strong antioxidative potential. However, as was already mentioned, AE might protect against pro-oxidative action of Cd at both levels of exposure not only directly, but also indirectly by decreasing the concentration of this xenobiotic in the liver [17]. 

Our previous findings [13] and the results of the current investigation show that the mechanisms of the hepatoprotective action of the extract may also involve its influence on the biosynthesis of MT in the liver, with which, in the form of complexes, Cd is retained in hepatocytes [26]. The decrease in the expression of MT1 and MT2 mRNA noted as a result of AE administration under the treatment with Cd allows us to conclude that the lower concentration of MT in the liver, previously described by us in these female rats [13], resulted from lower biosynthesis of this protein. Taking into account the fact that the intake of AE alone increased or had no influence on the concentration of MT (Table S3) [13] and the expression of MT1 and MT2 mRNA, the decline in MTs expression (and this protein concentration) due to the administration of AE under exposure to Cd might be mediated by the impact of the extract on the metabolism of this heavy metal. The positive correlations noted between MT1 and MT2 mRNA expression and Cd concentration in this organ confirm the validity of this reasoning. Although there was no relationship between the concentration of Cd in the diet and the extent of oxidative modifications of the evaluated macromolecules in the liver, the positive correlations between the concentration of Cd in this organ and the concentrations of LPO, 8-isoprostane, PC, and 3-NT indicate the connection between the intensity of the exposure and the extent of lipid peroxidation and oxidative modifications of proteins. The lack of dependence between Cd concentration in the liver and the concentration of 8-OHdG may be explained by the fact that the administration of AE under the exposure to the 5 mg Cd/kg diet provided better protection against oxidative modifications of DNA than at the lower treatment. 

Besides all the accomplishments of our research, we are also aware of its limitations. Since women are more susceptible to the toxic action of Cd (due to the influence of sex hormones on Cd metabolism and higher gastrointestinal absorption of this metal) than males [42], we have used a female rat model and therefore the conclusions from our study refer to the female liver. Thus, the further investigation evaluating the impact of AE on the male liver during low-level and moderate repeated intoxication with this xenobiotic is needed. Moreover, it should be taken into consideration that the effects of Cd and/or AE on the investigated parameters at particular time points (3, 10, 17, and 24 months) were not studied in the same rats, but in various subgroups (7–8 females) within particular experimental groups. These may explain why some effects of Cd and/or AE administration were observed or not at some time points or only a tendency to change was noted.

## 5. Conclusions

In summary, the findings of the present investigation indicate that the administration of AE during low-level and moderate long-term exposure to Cd provides entire protection against this metal-induced lipid peroxidation and DNA oxidation and almost completely prevents oxidative damage to proteins in the liver, and thus protects this organ from injury. The fact that the feeding with the 1 mg Cd/kg diet caused oxidative modification of the basic cellular macromolecules in the liver shows that even low intoxication with this xenobiotic creates a threat of this organ failure and more attention should be focused on environmental exposure to Cd as a risk factor of liver injury. Moreover, the findings of the current research together with the results of our previous studies in the same experimental model allow for the conclusion that the hepatoprotective effect of AE under intoxication with Cd may be, at least partially, explained by its ability to prevent the development of oxidative stress, and to decrease the biosynthesis of MT1 and MT2, as well as Cd accumulation in this organ. The present results taken together with our recently published findings on the beneficial influence of AE on the oxidative/reductive balance of the liver tissue and its morphology in the conditions of Cd intoxication, reflecting the environmental exposure of humans in industrialized and developing countries, seem to provide a solid foundation to recognize that the consumption of rich in bioactive substances, especially polyphenols, chokeberry products may be the promising natural way to protect the liver from the consequences of exposure to this metal. This is the most significant and practically useful finding of the current investigation. We are aware that the possibility of using chokeberry in the protection from unfavorable outcomes of exposure to Cd, including liver damage in humans, needs further investigation. Until now, we have evaluated and revealed the protective impact of AE regarding various effects of Cd action and in our opinion aronia products seem to be very promising in providing protection against negative outcomes of exposure to this xenobiotic. Moreover, the strong potential of the extract to counteract against pro-oxidative action of Cd disclosed by us may suggest that consumption of aronia products will also provide protection of the organism in the conditions of exposure to other pro-oxidants. 

## Figures and Tables

**Table 1:** Main and interactive effects of cadmium (Cd) and the extract from *Aronia melanocarpa* L. berries (AE) on the concentrations of lipid peroxides (LPO) and 8-isoprostane in the liver of female rats. ^1, 2.^

Parameter	Duration (Months)	1 mg Cd/kg Diet + AE				5 mg Cd/kg Diet + AE			
		Main Effect of Cd	Main Effect of AE	Main Effect of Cd + AE	Possible Character of Cd—AE Interaction	Main Effect of Cd	Main Effect of AE	Main Effect of Cd + AE	Possible Character of Cd—AE Interaction
**LPO**	**3**	NS	7.154 *	12.02 **	Antagonistic action −36 ^3^ < +33 + 0; −36 < +33	7.616 *	8.240 **	13.03 **	Antagonistic action 0 < +76 + 0; 0 < +76
	**10**	4.947 *	NS	10.59 **	Antagonistic action 0 < +63 + 0; 0 < +63	21.42 ***	NS	10.40 **	Antagonistic action 0 < +73 + 0; 0 < +73
	**17**	NS	NS	6.697 *	Antagonistic action 0 < +45 + 0; 0 < +45	NS	NS	5.846 *	Antagonistic action 0 < +42 + 0; 0 < +42
	**24**	34.12 ***	17.35 ***	NS	No interaction	22.76 ***	20.51 ***	NS	No interaction
**8-isoprostane**	**3**	9.396 **	13.69 ***	NS	No interaction	NS	12.84 **	NS	No interaction
	**10**	8.540 **	10.50 **	14.74 ***	Antagonistic action 0 < +36 + 0; 0 < +36	NS	4.952 *	10.24 **	Antagonistic action 0 < +14 + 0; 0 < +14
	**17**	NS	9.258 **	4.521 *	Antagonistic action −19 < +15 + 0; −19 < +15	NS	9.295 **	4.277 *	Antagonistic action 0 < +16 + 0; 0 < +16
	**24**	9.305 **	NS	NS	No interaction	4.618 *	4.326 *	NS	No interaction

^1^ The results of the ANOVA/MANOVA analysis are represented as *F* values and the level of statistical significance (*p*). *F* values having *p* < 0.05 were considered statistically significant (* *p* < 0.05, ** *p* < 0.01, *** *p* < 0.001). NS—not statistically significant (*p* > 0.05). ^2^ To assess the possible character of the interaction between Cd and AE, the effect of co-administration of Cd and AE was compared to the sum of the effects of separate action of Cd and AE (Cd + AE effect vs. Cd effect + AE effect). Cd effect, AE effect, and Cd + AE effect are expressed as percentage changes (+, increase; −, decrease) of a measured parameter in comparison to the control group.^3^ The values represent percentage changes.

**Table 2:** Main and interactive effects of cadmium (Cd) and the extract from *Aronia melanocarpa* L. berries (AE) on the concentration of protein carbonyl groups (PC) and 3-nitrotyrosine (3-NT) in the liver of female rats. ^1, 2.^

Parameter	Duration (Months)	1 mg Cd/kg Diet + AE				5 mg Cd/kg Diet + AE			
		Main Effect of Cd	Main Effect of AE	Main Effect of Cd + AE	Possible Character of Cd—AE Interaction	Main Effect of Cd	Main Effect of AE	Main Effect of Cd + AE	Possible Character of Cd—AE Interaction
**PC**	**3**	NS	5.909 *	7.451 *	0 = 0 + 0	NS	10.33 **	11.89 **	Antagonistic action 0 < +83 ^3^ + 0; 0 < +83
	**10**	39.66 ***	7.420 *	NS	No interaction	61.63 ***	5.823 *	NS	No interaction
	**17**	NS	12.12 **	NS	No interaction	NS	10.90 **	NS	No interaction
	**24**	NS	5.675 *	NS	No interaction	22.20 ***	11.07	NS	No interaction
**3-NT**	**3**	5.027 *	NS	21.31 ***	Antagonistic action −25 < 0 + 0; −25 < 0	NS	NS	13.75 ***	0 = 0 + 0
	**10**	8.709 **	29.24 ***	14.46 ***	Antagonistic action 0 < +49 + 0; 0 < +49	21.43 ***	25.04 ***	15.00 ***	Antagonistic action 0 < +89 + 0; 0 < +89
	**17**	5.516 *	6.181 *	4.872 *	Antagonistic action 0 < +52 + 0; 0 < +52	9.385 **	17.50 ***	14.69 ***	Antagonistic action 0 < +63 + 0; 0 < +63
	**24**	27.86 ***	19.81 ***	17.99 ***	Antagonistic action 0 < +2.2-fold + 0 0 < +2.2-fold	84.46 ***	82.46 ***	75.97 ***	Antagonistic action 0 < +2.3-fold + 0 0 < +2.3-fold

^1^ The results of the ANOVA/MANOVA analysis are represented as *F* values and the level of statistical significance (*p*). *F* values having *p* < 0.05 were considered statistically significant (* *p* < 0.05, ** *p* < 0.01, *** *p* < 0.001). NS—not statistically significant (*p* > 0.05). ^2^ To assess the possible character of the interaction between Cd and AE, the effect of co-administration of Cd and AE was compared to the sum of the effects of separate action of Cd and AE (Cd + AE effect vs. Cd effect + AE effect). Cd effect, AE effect, and Cd + AE effect are expressed as percentage changes or factors of changes (+, increase; -, decrease) of a measured parameter in comparison to the control group. In the case when the effects of Cd, AE, and Cd + AE were equal 0, the evaluation of the character of Cd—AE interaction was impossible. ^3^ The values represent percentage changes.

**Table 3:** Main and interactive effects of cadmium (Cd) and the extract from *Aronia melanocarpa* L. berries (AE) on the concentration of 8-hydroxy-2′-deoxyguanosine (8-OHdG) in the liver of female rats. ^1,^ ^2.^

Parameter	Duration (Months)	1 mg Cd/kg Diet + AE				5 mg Cd/kg Diet + AE			
		Main Effect of Cd	Main Effect of AE	Main Effect of Cd + AE	Possible Character of Cd—AE Interaction	Main Effect of Cd	Main Effect of AE	Main Effect of Cd + AE	Possible Character of Cd—AE Interaction
**8-OHdG**	**3**	-	-	-	-	-	-	-	-
	**10**	-	-	-	-	9.806 **	30.89 ***	20.72 ***	Antagonistic action 0 < +2.4-fold + 0; 0 < +2.4-fold
	**17**	NS	NS	NS	No interaction	NS	6.709 *	10.07 **	Antagonistic action 0 < +2-fold + 0; 0 < +2-fold
	**24**	6.002 *	13.94 ***	17.07 ***	Antagonistic action 0 < +2.3-fold + 0 0 < +2.3-fold	NS	35.73 ***	41.47 ***	Antagonistic action 0 > +2.4-fold + (−3.8-fold) 0 > −1.4-fold

^1^ The results of the ANOVA/MANOVA analysis are represented as *F* values and the level of statistical significance (*p*). *F* values having *p* < 0.05 were considered statistically significant (* *p* < 0.05, ** *p* < 0.01, *** *p* < 0.001). NS – not statistically significant (*p* > 0.05). ^2^ To assess the possible character of the interaction between Cd and AE, the effect of co-administration of Cd and AE was compared to the sum of the effects of separate action of Cd and AE (Cd + AE effect vs. Cd effect + AE effect). Cd effect, AE effect, and Cd + AE effect are expressed as factors of changes (+, increase; −, decrease) of a measured parameter in comparison to the control group.

**Table 4:** Main and interactive effects of cadmium (Cd) and the extract from *Aronia melanocarpa* L. berries (AE) on the expression of metallothionein 1 (MT1) and metallothionein 2 (MT2) in the liver of female rats. ^1, 2.^

Parameter	Duration (Months)	1 mg Cd/kg Diet + AE				5 mg Cd/kg Diet + AE			
		Main Effect of Cd	Main Effect of AE	Main Effect of Cd + AE	Possible Character of Cd—AE Interaction	Main Effect of Cd	Main Effect of AE	Main Effect of Cd + AE	Possible Character of Cd—AE Interaction
**MT1**	**3**	4.691 *	NS	24.36 ***	Antagonistic action 0 < +75 ^3^ + (+45); 0 < +120	15.42 ***	NS	25.920 ***	Antagonistic action +33 < +96 + (+45); +33 < +141
	**10**	NS	NS	7.940 ***	Antagonistic action 0 < +29 + 0; 0 < +29	24.79 ***	12.26 ***	30.44 ***	Antagonistic action 0 < +76 + 0; 0 < +76
	**17**	21.55 ***	23.17 ***	15.19 ***	Antagonistic action 0 < +56 + 0; 0 < +56	286.0 ***	168.0 ***	145.1***	Antagonistic action +26 < +2.9-fold + 0; +26 < +2.9-fold
	**24**	20.51 ***	9.010 **	6.319 **	Antagonistic action 0 < +48 + 0; 0 < +48	104.2 ***	70.80 ***	62.83 ***	Antagonistic action 0 < +2.2-fold + 0; 0 < +2.2-fold
**MT2**	**3**	NS	NS	19.82 ***	Antagonistic action 0 < +53 + (+37); 0 < +90	6.882 **	NS	33.25 ***	Antagonistic action 0 < +70 + (+37); 0 < +107
	**10**	14.44 ***	NS	38.58 ***	Antagonistic action 0 < +39 + (+17); 0 < +56	120.0 ***	23.49 **	85.02 ***	Antagonistic action +24 < +79 + (+17); +24 < +96
	**17**	32.08 ***	20.74 ***	7.220 **	Antagonistic action 0 < +50 + 0; 0 < +50	302.3 ***	160.3 ***	116.4 ***	Antagonistic action +28 < +2.7-fold + 0; +28 < +2.7-fold
	**24**	8.020 **	15.99 ***	4.805 *	Antagonistic action 0 < +23 + 0; 0 < +23	87.02 ***	65.29 ***	39.36 ***	Antagonistic action 0 < +73 + 0; 0 < +73

^1^ The results of the ANOVA/MANOVA analysis are represented as *F* values and the level of statistical significance (*p*). *F* values having *p* < 0.05 were considered statistically significant (* *p* < 0.05, ** *p* < 0.01, *** *p* < 0.001). NS—not statistically significant (*p* > 0.05). ^2^ To assess the possible character of the interaction between Cd and AE, the effect of co-administration of Cd and AE was compared to the sum of the effects of separate action of Cd and AE (Cd + AE effect vs. Cd effect + AE effect). Cd effect, AE effect, and Cd + AE effect are expressed as percentage changes or factors of changes (+, increase) of a measured parameter in comparison to the control group. ^3^ The values represent percentage changes.

## Supplementary Materials

The following are available online at https://www.mdpi.com/2072-6643/11/4/758/s1, Table S1: The influence of the extract from *Aronia melanocarpa* L. berries (AE) on the concentration of cadmium (Cd) in the blood (µg/L), liver (µg/g), and urine (µg/g of creatinine) of female rats, Table S2: The influence of the extract from *Aronia melanocarpa* L. berries (AE) on total antioxidative status (TAS), total oxidative status (TOS), oxidative stress index (OSI), and the concentration of hydrogen peroxide (H_2_O_2_) in the liver of female rats exposed to cadmium (Cd), Table S3: The influence of the extract from *Aronia melanocarpa* L. berries (AE) on the concentration of metallothionein (MT) (nmol/g) in the liver of female rats.

## Abbreviations

AE	extract from *Aronia melanocarpa* L. berries
ANOVA	one-way analysis of variance
ANOVA/MANOVA	two-way analysis of variance
bp	base pair
b.w.	body weight
Cd	cadmium
Cd^2+^	cadmium ion
CdCl_2_	cadmium chloride
Cu	copper
Cu^+^	copper(I) ion
cDNA	complementary deoxyribonucleic acid
CV	coefficient of variation
DEPC	diethyl dicarbonate
DNA	deoxyribonucleic acid
DPPH**^·^**	1,1-diphenyl-2-picrylhydrazyl
EIA	enzyme immunoassay
ELISA	enzyme-linked immunosorbent assay
Fe	iron
Fe^2+^	iron(II) ion
FR	free radicals
GAPDH	glyceraldehyde-3-phosphate dehydrogenase
HNE	4-hydroxyalkenal
HO**^·^**	hydroxyl radical
H_2_O_2_	hydrogen peroxide
LPO	lipid peroxides
MDA	malondialdehyde
mRNA	messenger ribonucleic acid
MT	metallothionein
MT1	metallothionein 1
MT2	metallothionein 2
-OH group	hydroxyl group
OSI	oxidative stress index
p	level of statistical significance
PC	protein carbonyl groups
r	correlation coefficient
RNA	ribonucleic acid
RNase-free DNase	ribonuclease-free deoxyribonuclease
ROS	reactive oxygen species
Real-Time PCR	real-time polymerase chain reaction
RT-PCR	reverse transcriptase polymerase chain reaction
SD	standard deviation
SE	standard error
TAS	total antioxidative status
TOS	total oxidative status
Trolox	6-hydroxy-2,5,7,8-tetramethylchroman-2-carboxylic acid
w/v	weight/volume
Zn	zinc
3-NT	3-nitrotyrosine
8-OHdG	8-hydroxy-2′-deoxyguanosine
