# Supplementary material for: Extract from Aronia melanocarpa L. Berries Protects Against Cadmium-induced Lipid Peroxidation and Oxidative Damage to Proteins and DNA in the Liver: A Study using a Rat Model of Environmental Human Exposure to this Xenobiotic
Source: Nutrients. 2019 Mar 31;11(4):758. doi: 10.3390/nu11040758 (PMC6520854; doi:10.3390/nu11040758)
Supplement: Supplementary file 1 [file nutrients-11-00758-s001.pdf]

Table S1. The influence of the extract from *Aronia melanocarpa* L. berries (AE) on the concentration of cadmium (Cd) in the blood (μg/L), liver (μg/g), and urine (μg/g of creatinine) of female rats. [1, 2, 3, 4]

Duration (Months)	1 mg Cd/kg Diet			5 mg Cd/kg Diet		
	Cd Concentration in the Cd_1 Group	Effect of Cd + AE	Effect of AE	Cd Concentration in the Cd_5 Group	Effect of Cd + AE	Effect of AE
			**Blood**			
**3**	0.1884 ± 0.0100*	↑ 2.7-fold	⇔	1.0236 ± 0.066***	↑ 12-fold	↙ 19%
**10**	0.1792 ± 0.0198*	↑ 2.1-fold	⇔	0.9394 ± 0.0439***	↑ 9.2-fold	↙ 15%
**17**	0.2425 ± 0.0167***	↑ 3.2-fold	⇔	1.0339 ± 0.0266***	↑ 12.5-fold	↙ 10%
**24**	0.2330 ± 0.0143**	↑ 2.6-fold	⇔	1.0467 ± 0.0508***	↑ 10.2-fold	↙ 11%
			**Liver**			
**3**	0.1447 ± 0.0093***	↑ 2.8-fold	↙ 33%	0.912 ± 0.053***	↑ 21-fold	↙ 18%
**10**	0.199 ± 0.028***	↑ 7.8-fold	⇔	1.617 ± 0.112***	↑ 62-fold	↙ 11%
**17**	0.211 ± 0.019***	↑ 14-fold	⇔	2.449 ± 0.178***	↑ 134-fold	↙ 24%
**24**	0.364 ± 0.025***	↑ 17-fold	↙ 37%	2.755 ± 0.089***	↑ 182-fold	↙ 10%
			**Urine**			
**3**	0.2184 ± 0.0081**	↑ 58%	⇔	0.5008 ± 0.0234***	↑ 4.4-fold	↗ 21%
**10**	0.1809 ± 0.0194**	↑ 47%	⇔	0.4002 ± 0.0480***	↑ 3.8-fold	↗ 25%
**17**	0.2096 ± 0.0215*	↑ 44%	⇔	0.4147 ± 0.0390***	↑ 3.9%	↗ 33%
**24**	0.2053 ± 0.0155*	↑ 56%	⇔	0.4104 ± 0.0198***	↑ 3.7-fold	↗ 22%

[1] The rats were given 0.1% aqueous AE and Cd in diet at the concentration of 1 or 5 mg/kg for 3–24 months. [2] Table presents Cd concentration and changes in its concentration ($p < 0.05$; ANOVA, Duncan's multiple range post hoc test) compared to the control group (* $p < 0.05$, ** $p < 0.01$, *** $p < 0.001$): a percentage or factor of increase (↑) and the appropriate group treated with Cd alone: a percentage increase (↗), decrease (↙), or lack of statistically significant change (⇔; $p > 0.05$) are indicated. Cd concentration in control groups (mean ± SE) ranged from 0.0691 ± 0.0079 to 0.0860 ± 0.0091 μg/L in the blood, 0.0137 ± 0.0015 to 0.0348 ± 0.0026 μg/g in the liver, and from 0.1304 ± 0.0080 to 0.1491 ± 0.0103 μg/g of creatinine in the urine. [3] Detailed data on the impact of AE on the concentration of Cd in the blood, liver, and urine of rats exposed to this heavy metal have already been published [17]. [4] The concentrations of Cd in the blood and urine (biomarkers of intoxication with this xenobiotic) of the rats exposed to the 1 mg Cd/kg diet (0.103–0.324 μg/L and 0.085–0.354 μg/g creatinine, respectively) and 5 mg Cd/kg diet (0.584–1.332 μg/L and 0.284–0.820 μg/g creatinine, respectively), alone or together with AE [17] were within the range of this metal concentrations noted in the blood and urine of the general population in industrialized and developing countries [5,9,10].

Table S2. The influence of the extract from *Aronia melanocarpa* L. berries (AE) on total antioxidative status (TAS), total oxidative status (TOS), oxidative stress index (OSI), and the concentration of hydrogen peroxide (H_2O_2) in the liver of female rats exposed to cadmium (Cd). [1, 2]

Duration (Months)	1 mg Cd/kg Diet			5 mg Cd/kg Diet		
	**Effect of Cd Alone**	**Effect of Cd + AE**	**Effect of AE**	**Effect of Cd Alone**	**Effect of Cd + AE**	**Effect of AE**
			**TAS**			
**3**	↔	↔	⬄	↔	↔	⬄
**10**	↓ 28%	↓ 30%	⬄	↓ 19%	↔	⬄
**17**	↓ 46%	↓ 31%	⬄	↔	↔	⬄
**24**	↔	↑ 18%	↗ 36%	↔	↑ 43%	↗ 54%
			**TOS**			
**3**	↔	↔	⬄	↔	↔	⬄
**10**	↔	↓ 38%	↙ 40%	↔	↔	↙ 19%
**17**	↔	↓ 29%	↙ 32%	↔	↔	↙ 20%
**24**	↔	↔	⬄	↑ 51%	↑ 36%	↙ 14%
			**OSI**			
**3**	↔	↔	⬄	↑ 17%	↔	↙ 14%
**10**	↑ 43%	↔	↙ 38%	↑ 30%	↔	↙ 26%
**17**	↑ 92%	↔	↙ 48%	↑ 21%	↔	↙ 26%
**24**	↔	↓ 23%	↙ 24%	↑ 61%	↔	↙ 41%
			**H_2O_2**			
**3**	↔	↓ 33%	↙ 38%	↔	↔	↙ 24%
**10**	↑ 75%	↔	↙ 47%	↑ 73%	↔	↙ 37%
**17**	↑ 3.2-fold	↔	↙ 4.7-fold	↑ 3.4-fold	↔	↙ 4.8-fold
**24**	↑ 2.8-fold	↔	↙ 2.8-fold	↑ 2.9-fold	↔	↙ 3.1-fold

[1] The rats were given 0.1% aqueous AE and Cd in diet at the concentration of 1 or 5 mg/kg for 3–24 months. [2] Table presents changes in Cd concentration ($p < 0.05$; ANOVA, Duncan's multiple range post hoc test) compared to the control group: a percentage or factor of increase (↑), decrease (↓), or lack of statistically significant change (↔; $p > 0.05$) and appropriate group treated with Cd alone: a percentage increase (↗), decrease (↙) or lack of statistically significant change (⬄; $p > 0.05$) are indicated.

Table S3. The influence of the extract from *Aronia melanocarpa* L. berries (AE) on the concentration of metallothionein (MT) (nmol/g) in the liver of female rats. [1, 2, 3]

Duration (Months)	1 mg Cd/kg Diet			5 mg Cd/kg Diet		
	MT Concentration in the Cd_1 Group	Effect of Cd + AE	Effect of AE	MT Concentration in the Cd_5 Group	Effect of Cd + AE	Effect of AE
**3**	51.96 ± 17.43***	↔	↙ 48%	59.83 ± 17.74***	↔	↙ 50%
**10**	55.75 ± 15.22***	↔	↙ 60%	62.07 ± 14.21***	↔	↙ 44%
**17**	61.77 ± 14.09***	↑ 27%	↙ 41%	69.96 ± 8.855***	↔	↙ 61%
**24**	63.40 ± 24.62***	↔	↙ 43%	79.85 ± 15.93***	↔	↙ 65%

[1] The rats were given 0.1% aqueous AE and Cd in diet at the concentration of 1 or 5 mg/kg for 3–24 months. [2] Table presents MT concentration and changes in its concentration ($p < 0.05$; ANOVA, Duncan's multiple range post hoc test) compared to the control group (*** $p < 0.001$): a percentage of increase (↑) or lack of statistically significant change (↔; $p > 0.05$) and the appropriate group treated with Cd alone: a percentage of decrease (↙) are indicated. MT concentration in the liver in the control group (mean ± SE) ranged from 23.90 ± 9.079 to 30.22 ± 11.81 nmol/g. [3] Detailed data on the impact of Cd and AE on the concentration of MT in the liver of rats exposed to this heavy metal have already been published [13].
